# Prediction Models for Individual-Level Healthcare Costs Associated with Cardiovascular Events in the UK

**DOI:** 10.1007/s40273-022-01219-6

**Published:** 2023-02-23

**Authors:** Junwen Zhou, Runguo Wu, Claire Williams, Jonathan Emberson, Christina Reith, Anthony Keech, John Robson, Kenneth Wilkinson, Jane Armitage, Alastair Gray, John Simes, Colin Baigent, Borislava Mihaylova

**Affiliations:** 1 Health Economics Research Centre, Nuffield Department of Population Health, University of Oxford, Old Road Campus, Headington, OX3 7LF Oxford UK; 2 Health Economics and Policy Research Unit, Wolfson Institute of Population Health, Queen Mary University of London, London, UK; 3 MRC Population Health Research Unit, Nuffield Department of Population Health, University of Oxford, Oxford, UK; 4 Clinical Trial Service Unit and Epidemiological Studies Unit, Nuffield Department of Population Health, University of Oxford, Oxford, UK; 5 NHMRC Clinical Trials Centre, University of Sydney, Sydney, Australia; 6 Clinical Effectiveness Group, Wolfson Institute of Population Health, Queen Mary University of London, London, UK; 7 Oxfordshire, UK

## Abstract

**Objectives:**

The aim of this study was to develop prediction models for the individual-level impacts of cardiovascular events on UK healthcare costs.

**Methods:**

In the UK Biobank, people 40–70 years old, recruited in 2006–2010, were followed in linked primary (*N* = 192,983 individuals) and hospital care (*N* = 501,807 individuals) datasets. Regression models of annual primary and annual hospital care costs (2020 UK£) associated with individual characteristics and experiences of myocardial infarction (MI), stroke, coronary revascularization, incident diabetes mellitus and cancer, and vascular and nonvascular death are reported.

**Results:**

For both people without and with previous cardiovascular disease (CVD), primary care costs were modelled using one-part generalised linear models (GLMs) with identity link and Poisson distribution, and hospital costs with two-part models (part 1: logistic regression models the probability of incurring costs; part 2: GLM with identity link and Poisson distribution models the costs conditional on incurring any). In people without previous CVD, mean annual primary and hospital care costs were £360 and £514, respectively. The excess primary care costs were £190 and £360 following MI and stroke, respectively, whereas excess hospital costs decreased from £4340 and £5590, respectively, in the year of these events, to £190 and £410 two years later. People with previous CVD had more than twice higher annual costs, and incurred higher excess costs for cardiovascular events. Other characteristics associated with higher costs included older age, female sex, south Asian ethnicity, higher socioeconomic deprivation, smoking, lower level of physical activities, unhealthy body mass index, and comorbidities.

**Conclusions:**

These individual-level healthcare cost prediction models could inform assessments of the value of health technologies and policies to reduce cardiovascular and other disease risks and healthcare costs. An accompanying Excel calculator is available to facilitate the use of the models.

**Supplementary Information:**

The online version contains supplementary material available at 10.1007/s40273-022-01219-6.

## Key Points for Decision Makers

**Table:** 

This is the first large observational cohort study examining the impact of cardiovascular events on costs of primary and hospital care in people without and with previous CVD.
The cost models enable individual-level cost estimates; an Excel calculator is available to facilitate their use.
These contemporary UK estimates can inform economic and policy assessments of the value of health technologies and policies to reduce cardiovascular disease risks as well as burden of disease analyses.

## Introduction

Cardiovascular disease (CVD) is the leading cause of global disease burden [1]. In the United Kingdom (UK), 7.6 million people are living with CVD, and half of the population are predicted to develop a CVD condition during their lifetime [2]. In 2015, CVD was estimated to incur £9 billion of healthcare costs in the UK, 4.7% of the total healthcare budget, and to cost society £19 billion [3, 4].

Decisions to adopt policies and interventions to reduce CVD risk require reliable estimates of the impact of CVD events on healthcare costs. However, few studies report comprehensive estimates. Previous studies in the UK focused on people with previous CVD [5–8] and on hospital care costs only [8–12] and were often limited in size [7–12]. Contemporary estimates of the impacts of CVD events on healthcare costs, particularly among people without previous CVD, and the impacts on primary care costs and over the long term are needed.

This study used the UK Biobank study data of 500,000 adults to quantify the immediate and long-term impact of incident CVD events on primary and hospital care costs in categories of people without and with previous CVD.

## Methods

### Study Population and Data

UK Biobank is a prospective cohort of > 500,000 adults from across England, Scotland and Wales aged 40–69 years at recruitment in 2006–2010. All UK Biobank participants with established linkage to primary (192,983 [38.5%] participants) or hospital care (501,807 [100%] participants) records were included; the lack of primary care data for research purposes for 61.5% of participants was due to lack of agreement with data providers. A small number of participants with end-stage renal disease at entry into UK Biobank were excluded as dialysis information was not included in the linked hospital data [13]. The linked healthcare data were used to identify the primary and hospital care records from participants’ entry to 31 March 2016 (the latest date all required linked data was available at 2019 datacut), death or loss to follow-up, whichever occurred first. Participants who had moved to primary care practices not contributing linked data had gaps in their registration records in the linked primary care data. The annual periods of participant data with such registration gaps were excluded from the primary care cost analysis.

### Adverse Events Identification

The first occurrences of four cardiovascular events after entry into UK Biobank: myocardial infarction (MI), stroke, coronary revascularization (CRV) and vascular death (VD) were the focus of the study. Three non-vascular events were also identified: incident diabetes, incident cancer and non-vascular death (NVD). Events were identified using the earliest date identified from the linked primary care, hospital admission and cancer and death registry records (see Supplement section 1 and Supplement table 1 of Online Resource 1 in the electronic supplementary material [ESM]).

### Costing Primary and Hospital Care

Three categories of primary care services recorded in the primary care records were costed: primary care consultations, diagnostic and monitoring tests, and prescription medications. Primary care consultations were costed using an average cost per general practice (GP) consultation of £29.01 based on the unit cost per minute by GP staff [14], duration of consultation by GP staff [15], and proportions of types of consultations by GP and practice nurse [16] (Supplement section 2 of Online Resource 1, see ESM). Diagnostic and monitoring tests were costed by identifying and costing categories of tests using the relevant average unit cost from the National Health Service (NHS) England reference cost [17] (Supplement table 2 of Online Resource 1, see ESM). Prescription medications were costed using the mean cost per prescription at paragraph level from NHS prescription cost analysis [18].

Hospital care services, identified in the hospital admission records, were costed by mapping the hospital inpatient episodes into Healthcare Resource Groups (HRGs), the standard grouping of clinically similar treatments using the HRG4+ reference costs grouper [19]. HRGs and the excess bed days were costed using the NHS England reference costs [17, 20].

All costs were inflated to year 2020 using the NHS cost inflation index [21].

### Statistical Analysis

Annual primary and hospital care costs were calculated over annual periods from recruitment into UK Biobank by summing up the costs incurred by each participant during each year of follow-up in the study. For the hospital admissions spanning more than 1 year of follow-up, a share of the total cost was allocated into each respective annual period based on the duration of hospital stay in that annual period. Separate statistical models were estimated for annual primary and annual hospital care costs.

The following participant characteristics at entry were considered for inclusion in the cost models: sex, ethnicity, quintile of Townsend deprivation index [22], smoking status, physical activity, diet quality, body mass index (BMI), low density lipoprotein (LDL) cholesterol, high density lipoprotein (HDL) cholesterol, serum creatinine, systolic blood pressure (SBP), diastolic blood pressure (DBP), antihypertensive treatment and histories of diabetes mellitus, severe mental illness or CVD. The history of CVD was specified as follows: prior myocardial infarction only, prior stroke only, prior peripheral arterial disease only, prior other coronary heart disease only (including acute rheumatic fever, chronic rheumatic heart diseases, hypertensive heart disease, angina pectoris, other acute ischaemic heart disease, chronic ischaemic heart disease, pulmonary heart disease and other form of heart disease); and prior events in two or more of the above categories. The following annually updated participant characteristics were also considered: current age, duration since first occurrences of cardiovascular events during follow-up and duration since incident diabetes or cancer. For each event of interest, duration since event was defined as a categorical variable with the following levels: no event; event in the annual period (same year); event in the previous annual period (1 year ago); event in the annual period two years ago (2 years ago); and event in an annual period three or more years ago (≥ 3 years ago). Missing data handling and covariate specification for modelling were specified (Supplement section 3 of Online Resource 1, see ESM) and interactions between co-occurring events during annual periods were assessed (Supplement section 4 of Online Resource 1, see ESM). Separate statistical models were estimated in participants without and with previous CVD at entry as preliminary analyses indicated important differences in impacts.

Annual primary care costs were modelled using generalized linear regression models (GLMs). Due to the high proportion of zero-cost observations (no hospital use in year) and highly skewed distribution for non-zero costs, annual hospital care costs were modelled using two-part models with the first part modelling the probability of having any costs using logistic regression, and the second part modelling the costs conditional on having incurred costs using GLMs (Supplement section 5 of Online Resource 1, see ESM). Six different GLMs using three distributions (Gaussian, Poisson, and Gamma) and two link functions (identity and natural log) were considered for the primary care costs models and for the second part of the hospital costs models. The selection of the most appropriate models was based on common specification tests, predictive performance, and parsimony (Supplement section 6 of Online Resource 1, see ESM). Cluster robust standard errors were estimated acknowledging the lack of independence between annual periods for the same participant. Stepwise bidirectional covariate selection (backward elimination and forward selection) was performed at the 1% level of statistical significance.

The mean excess annual costs associated with events in the UK Biobank were summarised using the estimated cost models with recycled prediction used for the hospital cost model (see further details in Supplement section 7 of Online Resource 1 [ESM]). The confidence intervals (CIs) for the estimated mean annual hospital costs for the reference individual were derived using a bootstrap approach with 1000 resamples with replacement from participant data.

Analyses were performed using R version 4.1.3.

## Results

501,807 UK Biobank participants (100%) with 3,798,324 annual periods of follow-up contributed to the hospital cost analyses, and 192,983 UK Biobank participants (38.5%) with 1,255,741 annual periods of follow-up contributed to the primary care cost analyses (Supplement figure 1 of Online Resource 1, see ESM). The average age at recruitment of participants contributing to the hospital cost analyses was 56.5 years (standard deviation: 8.1), 45.6% were men, and 57,271 (11.4%) were with previous CVD. The characteristics at baseline of participants contributing and not contributing to the primary care analyses, respectively, were similar (Supplement table 3 of Online Resource 1, see ESM). Participants with previous CVD were older, more likely to be men, of lower socioeconomic status, smokers, with lower level of physical activities, unhealthy diet, higher BMI, and higher proportion of disease histories such as hypertension, diabetes, cancer and severe mental illness (Table 1).

**Table 1:** Baseline characteristics of study participants

	Primary care costs analysis *Mean (SD) or n* (%)				Hospital care costs analysis *Mean (SD) or n* (%)			
	Without previous CVD (*n* = 168,205)		With previous CVD (*n* = 24,778)		Without previous CVD (*n* = 444,536)		With previous CVD (*n* = 57,271)	
Age (years)	56.1 (8.0)		60.3 (7.1)		56.0 (8.1)		60.4 (7.0)	
Male	73,573 (43.7)		14,084 (56.8)		194,979 (43.9)		33,729 (58.9)	
Ethnicity								
White	159,517 (94.8)		23,570 (95.1)		417,964 (94)		54,121 (94.5)	
Black	1851 (1.1)		222 (0.9)		7266 (1.6)		770 (1.3)	
South Asian	2905 (1.7)		495 (2)		6983 (1.6)		1058 (1.8)	
Other*	3170 (1.9)		355 (1.4)		9912 (2.2)		962 (1.7)	
Missing	762 (0.5)		136 (0.5)		2411 (0.5)		360 (0.6)	
Townsend socioeconomic deprivation								
Quintile 1 (least deprived)	63,113 (37.5)		8248 (33.3)		166,039 (37.4)		18,951 (33.1)	
Quintile 2	34,644 (20.6)		4880 (19.7)		89,211 (20.1)		10,939 (19.1)	
Quintile 3	27,656 (16.4)		3977 (16.1)		72,492 (16.3)		9019 (15.7)	
Quintile 4	24,526 (14.6)		3982 (16.1)		64,358 (14.5)		9155 (16.0)	
Quintile 5	18,029 (10.7)		3657 (14.8)		51,883 (11.7)		9138 (16.0)	
Missing	237 (0.1)		34 (0.1)		553 (0.1)		69 (0.1)	
Smoking								
Never	94,763 (56.3)		11,069 (44.7)		248,296 (55.9)		24,880 (43.4)	
Former smoker	55,607 (33.1)		10,654 (43)		147,781 (33.2)		24,998 (43.6)	
Current smoker	17,016 (10.1)		2874 (11.6)		45,979 (10.3)		6927 (12.1)	
Missing	819 (0.5)		181 (0.7)		2480 (0.6)		466 (0.8)	
Physical activity								
Low	24,777 (14.7)		4265 (17.2)		65,921 (14.8)		10,104 (17.6)	
Moderate	55,199 (32.8)		7676 (31)		146,146 (32.9)		17,677 (30.9)	
High	55,680 (33.1)		7468 (30.1)		145,192 (32.7)		16,776 (29.3)	
Missing	32,549 (19.4)		5369 (21.7)		87,277 (19.6)		12,714 (22.2)	
Diet quality								
Healthy	108,313 (64.4)		15,553 (62.8)		285,989 (64.3)		35,570 (62.1)	
Unhealthy	56,945 (33.9)		8643 (34.9)		149,077 (33.5)		20,166 (35.2)	
Missing	2947 (1.8)		582 (2.3)		9470 (2.1)		1535 (2.7)	
Body mass index (kg/m^2^)								
< 18.5	840 (0.5)		128 (0.5)		2364 (0.5)		253 (0.4)	
≥ 18.5, < 25	55,226 (32.8)		6035 (24.4)		148,846 (33.5)		13,352 (23.3)	
≥ 25, < 30	71,515 (42.5)		10,344 (41.7)		187,957 (42.3)		23,874 (41.7)	
≥ 30, < 35	28,779 (17.1)		5485 (22.1)		74,396 (16.7)		13,037 (22.8)	
≥ 35, < 40	7949 (4.7)		1802 (7.3)		20,645 (4.6)		4312 (7.5)	
≥ 40	2990 (1.8)		733 (3)		7871 (1.8)		1813 (3.2)	
Missing	906 (0.5)		251 (1)		2457 (0.6)		630 (1.1)	
LDL cholesterol (mmol/L)	3.6 (0.9)		3.1 (0.9)		3.6 (0.8)		3.1 (0.9)	
HDL cholesterol (mmol/L)	1.5 (0.4)		1.3 (0.4)		1.5 (0.4)		1.3 (0.4)	
Creatinine (umol/L)	71.5 (15.1)		76.5 (19.5)		71.5 (15.1)		77.0 (19.8)	
Systolic blood pressure (mmHg)	138.2 (18.7)		139.1 (19.0)		137.8 (18.6)		138.9 (18.9)	
Diastolic blood pressure (mmHg)	82.6 (10.1)		81.0 (10.4)		82.4 (10.1)		80.9 (10.5)	
On antihypertensive treatment	27,240 (16.2)		10,900 (44)		71,925 (16.2)		26,181 (45.7)	
Prior diabetes								
Type 1	926 (0.6)		558 (2.3)		2487 (0.6)		1378 (2.4)	
Type 2	7134 (4.2)		2694 (10.9)		19,075 (4.3)		6792 (11.9)	
Prior cancer	12,221 (7.3)		2420 (9.8)		32,712 (7.4)		5859 (10.2)	
Severe mental illness history	17,549 (10.4)		3374 (13.6)		36,082 (8.1)		6323 (11)	
Prior CVD								
No	168,205 (100)		0 (0)		444,536 (100)		0 (0)	
MI only	0 (0)		776 (3.1)		0 (0)		2070 (3.6)	
Stroke only	0 (0)		1991 (8)		0 (0)		5137 (9)	
PAD only	0 (0)		3473 (14)		0 (0)		6805 (11.9)	
Other CHD only^	0 (0)		12,642 (51)		0 (0)		28,969 (50.6)	
Two or more	0 (0)		5896 (23.8)		0 (0)		14,290 (25)	

Values are mean (SD) or number (%)

*CHD* coronary heart disease, *CVD* cardiovascular disease, *HDL* high-density lipoprotein, *LDL* low-density lipoprotein, *MI* myocardial infarction, *PAD* peripheral arterial disease, *SD* standard deviation

*Other ethnicity includes Chinese, Mixed, White and Black Caribbean, White and Black African, White and Asian, Any other mixed background and other ethnic group

^Other CHD includes acute rheumatic fever, chronic rheumatic heart diseases, hypertensive heart disease, angina pectoris, other acute ischaemic heart disease, chronic ischaemic heart disease, pulmonary heart disease and other form of heart disease

Over the average follow-up duration of 7.1 years, there were 6861 (1.4%) participants experiencing MIs, 5978 (1.2%) experiencing strokes, 8971 (1.8%) experiencing CRVs, 3193 (0.6%) died from vascular causes, and 11,699 (2.3%) died from nonvascular causes. Among the participants without previous diabetes or cancer at recruitment, 9696 (2.1%) were diagnosed with diabetes and 29,874 (6.7%) with cancer during follow-up, respectively. Compared with people without previous CVD, people with previous CVD had higher risks of adverse events (Supplement table 4 of Online Resource 1, see ESM).

The duration of follow-up was similar for both primary and hospital care costs analyses and across people without and with previous CVD. From the annual follow-up periods of participants with any linked primary care data, 13.7% included registration gaps in their linked primary care data and were excluded from analysis. Participants had on average 5.5 primary care consultations, 3.3 diagnostic and monitoring tests and 21.8 prescription medications per year, at an overall primary care cost of £409 per year (consultations £160; tests £31; medications £218); and 0.4 hospital admissions per year at an overall hospital inpatient cost of £583. Compared with people without previous CVD, people with previous CVD had higher rates of primary care consultations, diagnostic and monitoring tests, prescription medications and hospital admissions, and higher costs of primary (£360 vs £746) and hospital care (£514 vs £1131) (Supplement table 4 of Online Resource 1, see ESM).

**Fig. 1:**
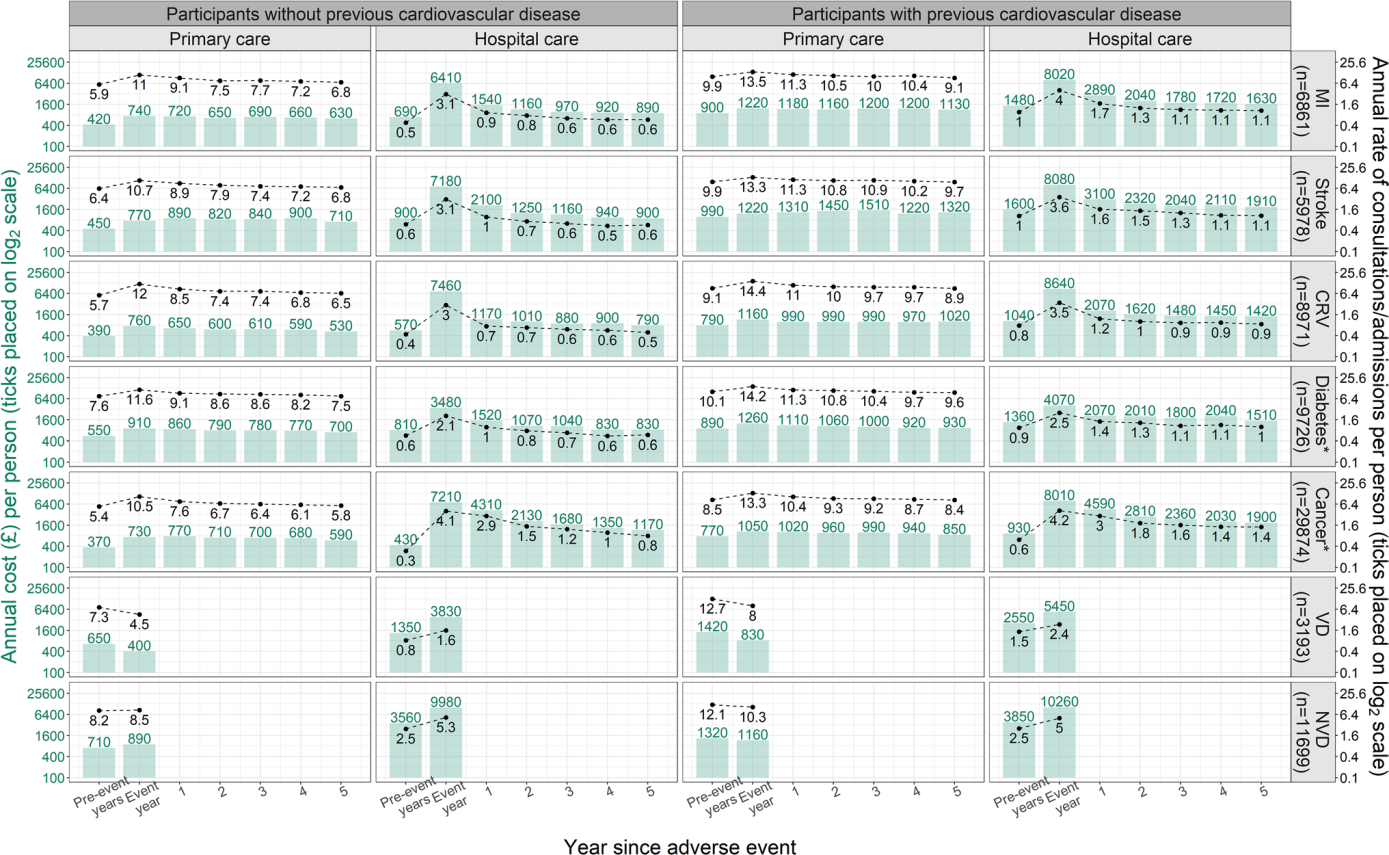
Annual costs (£) and rates of healthcare use by time since adverse event. In the figure, mean annual costs are summarised for UK Biobank participants experiencing the respective events. Annual periods are defined from date of participant’s entry into UK Biobank. ‘Event year’ corresponds to the annual periods with the respective event, ‘Pre-event years’ corresponds to annual periods prior to annual periods with respective events. Thereafter, data is presented for each subsequent annual period up to 5 years following the annual periods with events. Total number of first events in the study period were presented. For numbers of event in the separate analyses, please refer to Supplement table 4 of Online Resource 1 in the electronic supplementary material. *Incident cases only; *CRV* coronary revascularization, *MI* myocardial infarction, *NVD* non-vascular death, *VD* vascular death

Among participants who experienced MI, stroke, CRV, diabetes or cancer, annual primary care consultation rates and annual hospital admission rates peaked in the year of the event, then decreased over the subsequent years. The annual costs had a similar pattern across event types, except that the annual primary care costs continued to increase after the year of stroke. Among participants who died, the rates of primary care consultations and costs of primary care were generally lower in the year of death, whereas the rates and costs of hospital admissions were higher. Compared with participants without previous CVD, participants with previous CVD had higher annual primary care consultations rates and hospital admission rates, and higher annual primary care and hospital care costs (Fig. 1).

Following statistical model selection, primary care costs were assessed using one-part generalised linear models (GLMs) with Poisson distribution and identity link function (Table 2), and hospital care costs were assessed using two-part models (part 1: logistic regression models probability of incurring costs; part 2: GLM with Poisson distribution and identity link function models costs conditional on incurring any) (Table 3). In the final estimation models, the annual primary and hospital care costs (£) were 262 (95% CI 254–270) and 244 (95% CI 237–251), respectively, for the reference individual in models for people without previous CVD (a female aged 60 years, of white ethnicity, quintile 3 Townsend socioeconomic deprivation, never smoked, moderate physical activities, healthy diet quality and normal BMI level (18.5–25 kg/m^2^), with LDL cholesterol of 3.6 mmol/L, HDL cholesterol of 1 mmol/L, creatinine of 81.5 umol/L, SBP of 140 mmHg, DBP of 80 mmHg, not on antihypertensive treatments, and without histories of severe mental illness, cancer, diabetes, without incident cardiovascular or other studied events). The reference annual primary and hospital care costs (£) were 302 (258–345) and 376 (332–419), respectively, in models for people with previous CVD with the above characteristics except having history of MI alone at baseline. An example of how to calculate the annual costs using the models is provided in Supplement section 8 of Online Resource 1 (see ESM) and an Excel calculator, including variance-covariance matrices of the estimated models, accompanies the paper to facilitate the use of the models (Online Resource 2, see ESM).

**Table 2:** Annual primary care costs (£) models: Generalised linear models with Poisson distribution and identity link function

Covariate	Participants without previous CVD	Participants with previous CVD
	Mean (SE) ^a^	Mean (SE) ^a^
Intercept	262 (4)	302 (22)
*Baseline characteristics*		
Male (ref: female)	− 51 (3)	− 50 (13)
Ethnicity (ref: white)		
Black	− 3 (11)	61 (127)
South Asian	57 (9)	24 (36)
Others*	− 3 (2)	− 107 (24)
Townsend socioeconomic deprivation (ref: Quintile 3)		
Quintile 1 (least deprived)	− 5 (1)	6 (17)
Quintile 2	4 (2)	25 (17)
Quintile 4	6 (3)	49 (21)
Quintile 5	50 (6)	132 (34)
Smoking (ref: never)		
Former smoker	22 (2)	^b^
Current smoker	50 (5)	^b^
Physical activity (ref: moderate)		
Low	30 (5)	117 (21)
High	− 2 (2)	− 8 (10)
Missing	32 (4)	96 (21)
BMI (ref: ≥ 18.5, < 25)		
< 18.5	36 (15)	182 (107)
≥ 25, < 30	3 (2)	8 (11)
≥ 30, < 35	31 (4)	58 (16)
≥ 35, < 40	91 (9)	194 (46)
≥ 40	132 (13)	329 (44)
LDL cholesterol (centred at 3.6; per 1 mmol/L)	− 6 (2)	^b^
Natural logarithm of HDL cholesterol (lnmmol/L)	− 32 (6)	^b^
Systolic blood pressure (centred at 140; per 20 mmHg)	^b^	− 20 (6)
On antihypertensive treatment (ref: no)	127 (5)	93 (14)
Severe mental illness history (ref: no)	152 (6)	280 (31)
Prior type 1 diabetes (ref: no)	643 (45)	731 (78)
Prior CVD (ref: MI only)		
PAD only	NA	66 (19)
Stroke only	NA	124 (37)
Other CHD only^	NA	84 (17)
Two or more	NA	221 (22)
*Time-updated characteristics*		
Current age (centred at 60; per 10 years)	57 (2)	62 (7)
Incident MI (ref: no)	194 (24)	231 (54)
Incident stroke (ref: no)	362 (56)	428 (82)
Incident CRV (ref: no)		
Same year	391 (40)	233 (33)
≥ 1 year ago	69 (15)	10 (26)
Diabetes (ref: no)		
< 10 years ago	360 (11)	343 (24)
≥ 10 years ago	560 (19)	568 (39)
Cancer (ref: no)		
< 5 years	357 (11)	236 (25)
≥ 5, < 10 years	159 (13)	123 (28)
≥ 10 years ago	77 (6)	^c^
VD (ref = no)	− 95 (30)	− 16 (91)
NVD (ref = no)	389 (37)	198 (65)
*Interactions*		
Any incident MI and same year CRV (ref = no)	− 226 (47)	^b^
Any incident MI and same year VD (ref = no)	^b^	− 500 (109)
Any incident stroke and same year VD (ref = no)	− 277 (86)	− 439 (133)
< 5 years cancer and same year NVD (ref = no)	− 245 (47)	^b^

Costs in years with administrative censoring at end of study follow-up were adjusted by including a further covariate of proportion of year not observed (not shown)

For an example of how to use this model, see Supplement section 8 of Online Resource 1 in the electronic supplementary material

*BMI* body mass index, *CHD* coronary heart disease, *CRV* coronary revascularization, *CVD* cardiovascular disease, *HDL* high density lipoprotein, *LDL* low density lipoprotein, *MI* myocardial infarction, *NA* not applicable, *NVD* non-vascular death, *PAD* peripheral arterial disease, *SE* standard error, *VD* vascular death

*Other ethnicity includes Chinese, Mixed, White and Black Caribbean, White and Black African, White and Asian, any other mixed background and other ethnic group

^Other CHD includes acute rheumatic fever, chronic rheumatic heart diseases, hypertensive heart disease, angina pectoris, other acute ischaemic heart disease, chronic ischaemic heart disease, pulmonary heart disease and other form of heart disease

^a^The interpretation and use of model coefficients is as for any linear regression model. The intercept represents the annual cost for an individual in the reference categories of all covariates. All other coefficients represent the additional annual costs for an individual in the respective category compared with the reference category

^b^Covariate or interaction term excluded during the selection procedure (not statistically significant)

^c^Same as the preceding temporal category

**Table 3:** Annual hospital care costs (£) models: Two-part models (Part 1: Logistic regression model; Part 2: Generalised linear model with Poisson distribution and identity link function)

Covariate	Participants without previous CVD				Participant with previous CVD			
	Part 1: Likelihood of incurring cost		Part 2: Cost, if any incurred		Part 1: Likelihood of incurring cost		Part 2: Cost, if any incurred	
	OR (95% CIs) ^a^		Mean (SE) ^a^		OR (95% CIs) ^a^		Mean (SE) ^a^	
Intercept	0.13 (0.13–0.13)		2102 (23)		0.19 (0.18–0.21)		2326 (119)	
*Baseline characteristics*								
Male (ref: female)	0.92 (0.91–0.93)		− 65 (14)		0.87 (0.84–0.89)		− 125 (54)	
Ethnicity (ref: white)								
Black	1.04 (1–1.08)		− 117 (68)		1.06 (0.97–1.16)		− 412 (128)	
South Asian	1.14 (1.1–1.18)		− 168 (48)		1.21 (1.12–1.31)		− 426 (102)	
Others*	1.03 (1–1.06)		− 165 (49)		1.09 (1.01–1.19)		− 246 (145)	
Townsend socioeconomic deprivation (ref: Quintile 3)								
Quintile 1 (least deprived)	0.95 (0.94–0.96)		− 81 (20)		0.91 (0.89–0.94)		^b^	
Quintile 2	0.99 (0.98–1.01)		− 55 (22)		0.95 (0.92–0.99)		^b^	
Quintile 4	1.07 (1.05–1.08)		24 (27)		1.06 (1.02–1.1)		^b^	
Quintile 5	1.17 (1.15–1.19)		94 (27)		1.15 (1.11–1.19)		^b^	
Smoking (ref: never)								
Former smoker	1.11 (1.1–1.12)		40 (15)		1.06 (1.04–1.09)		8 (45)	
Current smoker	1.2 (1.18–1.22)		183 (24)		1.13 (1.09–1.17)		276 (85)	
Physical activity (ref: moderate)								
Low	1.1 (1.09–1.12)		110 (23)		1.25 (1.21–1.29)		415 (74)	
High	1.07 (1.05–1.08)		− 12 (16)		1.04 (1.02–1.07)		−13 (46)	
Missing	1.14 (1.13–1.16)		72 (20)		1.17 (1.13–1.2)		156 (51)	
Unhealthy diet (ref: healthy diet)	1.06 (1.05–1.07)		^b^		1.06 (1.04–1.09)		^b^	
Body mass index (kg/m^2^) (ref: ≥ 18.5, < 25)								
< 18.5	1.13 (1.06–1.2)		298 (169)		1.43 (1.23–1.66)		1007 (796)	
≥ 25, < 30	1.12 (1.11–1.13)		68 (16)		1.04 (1.01–1.07)		16 (50)	
≥ 30, < 35	1.24 (1.23–1.26)		239 (20)		1.14 (1.1–1.17)		177 (58)	
≥ 35, < 40	1.36 (1.33–1.39)		451 (35)		1.21 (1.16–1.27)		381 (87)	
≥ 40	1.51 (1.46–1.56)		649 (66)		1.34 (1.26–1.42)		840 (178)	
LDL cholesterol (centred at 3.6; per 1 mmol/L)	0.97 (0.96–0.97)		− 36 (8)		^b^		^b^	
Natural logarithm of HDL cholesterol (lnmmol/L)	0.86 (0.84–0.88)		^b^		^b^		^b^	
Natural logarithm of creatinine centred at 4.4; per 0.2 lnumol/L	0.98 (0.98–0.99)		^b^		1.02 (1.01–1.04)		107 (24)	
Systolic blood pressure (centred at 140; per 20 mmHg)	0.93 (0.93–0.94)		^b^		0.95 (0.94–0.96)		^b^	
Diastolic blood pressure (centred at 80; per 10 mmHg)	1.02 (1.01–1.02)		^b^		^b^		^b^	
On antihypertensive treatment (ref: no)	1.14 (1.13–1.16)		141 (20)		1.11 (1.09–1.14)		^b^	
Severe mental illness history (ref: no)	1.43 (1.41–1.45)		193 (25)		1.39 (1.34–1.43)		227 (66)	
Prior type 1 diabetes (ref: no)	1.83 (1.74–1.93)		702 (119)		1.69 (1.58–1.82)		792 (148)	
Prior CVD (ref: MI only)								
PAD only	NA		NA		1.19 (1.12–1.27)		498 (122)	
Stroke only	NA		NA		1.11 (1.04–1.19)		113 (118)	
Other CHD only^	NA		NA		1.27 (1.2–1.34)		105 (106)	
Two or more	NA		NA		1.43 (1.35–1.52)		381 (114)	
*Time-updated characteristics*								
Current age (centred at 60; per 10 years)	1.38 (1.37–1.39)		173 (9)		1.25 (1.23–1.27)		121 (31)	
Incident MI (ref: no)								
Same year	47.09 (38.69–57.32)		3054 (167)		47.33 (33.93–66.02)		3965 (241)	
1 year ago	1.76 (1.59–1.95)		670 (153)		1.71 (1.52–1.92)		1011 (303)	
2 years ago	1.44 (1.29–1.61)		304 (117)		1.28 (1.17–1.41)		696 (171)	
≥ 3 years ago	1.35 (1.22–1.49)		^c^		^c^		^c^	
Incident stroke (ref: no)								
Same year	47.08 (41.91–52.9)		4485 (142)		46.65 (36.98–58.85)		4591 (208)	
1 year ago	2.58 (2.39–2.8)		2192 (296)		2.19 (1.96–2.46)		1561 (260)	
2 years ago	1.78 (1.62–1.95)		833 (137)		1.52 (1.38–1.67)		^c^	
≥ 3 years ago	1.49 (1.37–1.61)		^c^		^c^		^c^	
Incident CRV (ref: no)								
Same year	^d^		5186 (114)		^d^		5117 (146)	
1 year ago	1.66 (1.53–1.81)		137 (82)		1.54 (1.41–1.68)		599 (192)	
2 years ago	1.51 (1.37–1.65)		^c^		1.32 (1.23–1.41)		4 (110)	
≥ 3 years ago	1.32 (1.22–1.43)		^c^		^c^		^c^	
Diabetes (ref: no)								
< 10 years ago	1.36 (1.33–1.39)		274 (35)		1.36 (1.32–1.41)		408 (80)	
≥ 10 years ago	1.2 (1.16–1.23)		158 (51)		1.22 (1.17–1.28)		^c^	
Cancer (ref: no)								
Same year	40.92 (39.33–42.58)		5380 (56)		24.59 (22.17–27.27)		5160 (150)	
1 year ago	6.04 (5.87–6.21)		4620 (86)		3.81 (3.55–4.08)		3475 (181)	
2 years ago	3.03 (2.94–3.12)		2332 (88)		2.39 (2.23–2.56)		1863 (172)	
3 years ago	2.46 (2.38–2.54)		1899 (89)		2.09 (1.95–2.25)		1601 (176)	
4 years ago	2.23 (2.16–2.31)		1502 (86)		1.92 (1.79–2.07)		947 (75)	
≥ 5 years ago	1.69 (1.66–1.72)		1159 (38)		1.6 (1.54–1.65)		^c^	
VD (ref = no)	2.32 (2.03–2.64)		4318 (491)		2.38 (2.07–2.74)		4749 (420)	
NVD (ref = no)	11.4 (10.69–12.16)		6792 (145)		9.1 (7.97–10.38)		6412 (260)	
*Event interactions*								
Same year MI and same year CRV (ref = no)	^d^		− 3848 (227)		^d^		− 3358 (364)	
Same year MI and same year VD (ref = no)	0.03 (0.02–0.04)		− 4694 (670)		0.02 (0.01–0.03)		− 4874 (722)	
Same year stroke and same year VD (ref = no)	0.22 (0.16–0.32)		− 4171 (685)		0.09 (0.06–0.14)		− 4308 (691)	
Same year cancer and same year NVD (ref = no)	0.35 (0.26–0.47)		− 1725 (291)		0.37 (0.21–0.66)		− 1529 (617)	

Costs in years with administrative censoring at end of study follow-up were adjusted by including a further covariate of proportion of year not observed (not shown)

To predict the annual costs using the two-part model, please follow the following steps (see also an example in Supplement section 8 of Online Resource 1 in the electronic supplementary material): (1) predict the odds of incurring any costs in the year (${\text{Odds}}_{{{\text{P}}1}}$) from the first part: ${\text{Odds}}_{{{\text{P}}1}} = {\text{exp}}^{{\ln \left( {{\text{Intercept}}} \right) + \mathop \sum \nolimits_{1}^{n} ({\text{ln}}({\text{OR}}_{i} ) \times X_{i} )}}$, (2) predict the annual costs assuming such were incurred in the year (${\text{Cost}}_{{{\text{P}}2}}$) from the second part: ${\text{Cost}}_{{{\text{P}}2}} = {\text{Intercept}} + \mathop \sum \nolimits_{1}^{n} ({\text{Mean}}_{i} \times X_{i} )$, (3) calculate the predicted annual costs using this formula: [${\text{Odds}}_{{{\text{P}}1}}$/(${\text{Odds}}_{{{\text{P}}1}}$+1)] × ${\text{Cost}}_{{{\text{P}}2}}$ where $X_{i}$ is the value of the *i*th covariate (excluding the intercept term)

*CHD* coronary heart disease, *CI* confidence interval, *CRV* coronary revascularization, *CVD* cardiovascular disease, *HDL* high-density lipoprotein, *LDL* low-density lipoprotein, *MI* myocardial infarction, *NA* not applicable, *NVD* non-vascular death, *OR* odds ratio, *PAD* peripheral arterial disease, *SE* standard error, *VD* vascular death

*Other ethnicity includes Chinese, Mixed, White and Black Caribbean, White and Black African, White and Asian, any other mixed background and other ethnic group

^Other CHD includes acute rheumatic fever, chronic rheumatic heart diseases, hypertensive heart disease, angina pectoris, other acute ischaemic heart disease, chronic ischaemic heart disease, pulmonary heart disease and other form of heart disease

^a^The intercept terms represent the respective values for an individual in the reference categories of covariates (odds for part 1 model and cost for part 2 model); other coefficients represent the added effect for that category of the covariate compared with the reference category (odds ratio for part 1 model and additional cost for part 2 model)

^b^Covariate was excluded during the selection procedure (not statistically significant)

^c^Same as the preceding temporal category

^d^Incurring cost is certain in annual periods with CRV

In participants without previous CVD, the excess primary and hospital care costs (£) in the year of cardiovascular event, estimated by the respective models, were 190 (95% CI 150–240) and 4340 (4050–4640) for MI, 360 (250–470) and 5590 (5340–5840) for stroke, 390 (310–470) and 7140 (6920–7360) for CRV, respectively (Table 4). In participants with previous CVD, the excess costs were higher: 230 (130–340) and 5610 (5170–6040) for MI, 430 (270–590) and 6170 (5780–6560) for stroke, 230 (170–300) and 7200 (6920–7490) for CRV. VD was only associated with excess hospital costs (£), which were 1560 (1240–1880) and 2720 (2280–3160) in participants without and with previous CVD (Table 4). Overall, the excess costs in the year of co-occurrence of MI and CRV, MI and VD and stroke and VD were lower than the sum of individual excess costs for respective events (Tables 2, 3). The excess primary care costs remained relatively stable after cardiovascular events, except for CRV, whereas the excess primary care costs decreased in the years following CRV (Table 4).

**Table 4:** Excess annual healthcare costs (£) associated with cardiovascular and non-vascular events

Event (reference: no)	Participants without previous CVD		Participants with previous CVD	
	Primary care cost (95% CI)	Hospital care cost (95% CI)	Primary care cost (95% CI)	Hospital care cost (95% CI)
*Cardiovascular events*				
Myocardial infarction				
Same year	190 (150–240)	4340 (4050–4640)	230 (130–340)	5610 (5170–6040)
1 year ago	^a^	370 (280–450)	^a^	710 (460–970)
2 years ago	^a^	190 (120–260)	^a^	370 (240–500)
≥ 3 years ago	^a^	160 (100–220)	^a^	^a^
Stroke				
Same year	360 (250–470)	5590 (5340–5840)	430 (270–590)	6170 (5780–6560)
1 year ago	^a^	1060 (870–1240)	^a^	1180 (940–1430)
2 years ago	^a^	410 (330–500)	^a^	810 (610–1010)
≥ 3 years ago	^a^	310 (240–390)	^a^	^a^
Coronary revascularization				
Same year	390 (310–470)	7140 (6920–7360)	230 (170–300)	7200 (6920–7490)
1 year ago	70 (40–100)	210 (160–270)	10 (−40 to 60)	480 (330–630)
2 years ago	^a^	170 (120–220)	^a^	160 (80–250)
≥ 3 years ago	^a^	120 (80–170)	^a^	^a^
Vascular death	−100 (−150 to ﻿−40)	1560 (1240–1880)	−20 (−190 to 160)	2720 (2280–3160)
*Non-vascular events*				
Diabetes				
< 10 years ago	360 (340–380)	160 (140–170)	340 (300–390)	310 (260–370)
≥ 10 years ago	560 (520–600)	90 (60–110)	570 (490–650)	240 (190–300)
Cancer				
Same year	360 (340–380)	6150 (6050–6250)	240 (190–290)	6240 (5970–6520)
1 year ago	^a^	2970 (2890–3060)	^a^	2740 (2520–2950)
2 years ago	^a^	1160 (1100–1230)	^a^	1340 (1170–1510)
3 years ago	^a^	850 (800–900)	^a^	1090 (930–1250)
4 years ago	^a^	670 (620–720)	^a^	750 (670–840)
≥ 5, < 10 years ago	160 (130–180)	410 (400–430)	120 (70–180)	600 (540–660)
≥ 10 years ago	80 (70–90)	^a^	^a^	^a^
Non-vascular death	390 (320–460)	5350 (5130–5560)	200 (70–330)	6040 (5630–6460)

Estimates adjusted for patient sociodemographic and clinical characteristics and rounded to UK£10

*CI* confidence interval, *CVD* cardiovascular disease, *MI* myocardial infarction

^a^Excess annual cost remains as in preceding annual period (e.g. £190 excess annual primary care costs in each year following MI)

The estimated excess costs associated with incident diabetes, cancer and NVD were also generally larger among people with previous CVD (Table 4). Unlike most other events, the excess primary care costs associated with incident diabetes were somewhat larger in years following diagnosis. Substantial excess healthcare costs were noted in years with incident cancers and in subsequent years. Participant characteristics associated with higher primary care cost included older age, female sex, south Asian ethnicity, higher socioeconomic deprivation, smoking, low level of physical activity, unhealthy BMI, and morbidity, including antihypertensive treatment, severe mental illness, or prior diabetes or CVD (Table 2). Similar characteristics, except south Asian ethnicity, were also associated with higher hospital care costs (Table 3).

## Discussion

This large observational cohort study quantified the temporal impacts of cardiovascular disease events on primary and hospital care costs for people with different characteristics. Overall, both primary and hospital care costs increased substantially in years with events. The excess hospital care costs decreased substantially in the following years. Excess primary care costs were relatively lower than excess hospital costs in the years of events and remained largely stable, contributing a growing proportion of total excess health care costs.

Comparisons with previous work on excess healthcare costs are difficult due to the focus on particular patient populations in these studies, such as patients with chronic kidney disease (CKD) [10] or diabetes [11], and on different healthcare systems [23, 24]. To the extent to which the results can be compared, our estimates of excess costs were either somewhat larger [5, 9] or similar [6] in size to previous estimates. Our excess primary and hospital care costs associated with adverse events were comparable to estimates presented in two previous studies among patients with previous CVD [6, 7].

Our study provides much-needed contemporary evidence for long-term primary and hospital care costs associated with cardiovascular events, with the personalized cost models allowing individual-level estimates, and the estimates of average excess costs facilitating cost estimates in the absence of individual-level data. These estimated costs can inform economic and policy assessments of the value of health interventions to reduce CVD risk and burden-of-illness cost analyses. In particular, our results can be used to inform cost-effectiveness assessments of cardiovascular prevention treatments such as lipid or blood pressure management therapies, which are analysed using models including cardiovascular events studied in the present study. The individual-level predictions from the cost models or estimated average excess costs associated with particular events can directly inform healthcare costs in such analyses. In addition to morbidities such as cardiovascular disease, diabetes, and cancer, our study also indicates further key individual factors contributing to primary and hospital care costs, such as smoking, obesity and low physical activity, which could be targeted to alleviate demand and costs of healthcare services.

Our study has important methodological strengths, being based on a large observational UK cohort, with reliable participant data at recruitment and comprehensive routine primary and hospital care data during follow-up. Because of participant selection, the more customary trial-based analyses [8–12] are less likely to reflect typical healthcare consumption patterns than cohort studies and trials also rarely collect primary care data. Previous cohort studies [7, 25] had limited sample sizes and restricted their analyses to the excess healthcare costs after particular events. Two large retrospective studies [5, 6] used Clinical Practice Research Datalink (CPRD) data but assessed excess healthcare costs by simple subtraction of the costs in the years after events with the costs in the years before events without considering participants’ characteristics or cost trajectories. Conversely, we report models of annual healthcare costs including a range of patient characteristics and show that, while the excess annual primary care costs of cardiovascular events are similar across categories of patients (i.e. linear primary care cost models), the excess annual hospital inpatient costs vary (i.e. non-linear two-part hospital cost models) with, for example, female sex and higher age conferring higher odds of incurring costs and, therefore, higher excess hospital costs (Supplement table 5 of Online Resource 1, see ESM). Finally, we directly addressed the issue of co-occurrence of cardiovascular events which was not considered in previous studies, and report that the impact of co-occurring cardiovascular events on healthcare costs was not a simple addition of separate event impacts.

Our study has some potential limitations. Firstly, our study population was not representative of the general UK population. Although the detailed cost models adjust for a range of individual-level characteristics, including socio-economic deprivation, and are expected to perform well across broader categories of people, UK Biobank participants were 40–70 years old at recruitment and aged up to around 80 years during study follow-up. Therefore, further research to assess excess costs associated with adverse events in younger people as well as in the elderly will be helpful. Secondly, we excluded a small number of participants with end-stage renal disease due to unavailability of dialysis care data. Thirdly, we did not have data on outpatient hospital care use and, therefore, are likely underestimating total excess healthcare costs. However, the excess outpatient hospital care costs for cardiovascular events are comparatively small in the UK. For example, in a study by Luengo-Fernandez et al. [26], the healthcare costs for cardiovascular disease in the UK included hospital inpatient care costs (76%), drug prescriptions (18%) and primary care consultations (4%), with < 2% contributed by other health care including outpatient hospital care. Finally, the primary care cost models were estimated on the subset of UK Biobank participants with linked primary care data. The availability of linked primary care data was, however, independent of patient and practice characteristics and unlikely to have led to biases in estimated costs.

## Conclusions

This large observational cohort study reports contemporary estimates of temporal impacts of cardiovascular events on annual primary care and hospital admission costs in people without and with previous CVD in the UK. The study findings and accompanying personalized cost prediction models should aid health-care planning and commissioning of cardiovascular disease treatments and prevention interventions to reduce CVD risk. An accompanying Excel calculator is available to facilitate the use of the models.

## Supplementary Information

Below is the link to the electronic supplementary material.

Supplementary file1 (PDF 1403 KB)

Supplementary file2 (XLSX 208 KB)

## Declarations

### Data availability statement

Data may be obtained from a third party and are not publicly available. The datasets used in the current study are available from UK Biobank (https://www.ukbiobank.ac.uk/). Researchers can apply to use the UK Biobank resource.
