# Supplementary material for: Prediction Models for Individual-Level Healthcare Costs Associated with Cardiovascular Events in the UK
Source: Pharmacoeconomics. Author manuscript; Available in PMC 2023 May 1. (PMC10085892; doi:10.1007/s40273-022-01219-6)
Supplement: Supplementary file [file EMS170796-supplement-Supplementary_file.pdf]

Prediction models for individual-level healthcare costs associated with cardiovascular events in the UK

Supplemental material

Table of contents

Supplement sections ... 2

Supplement section 1. Adverse events identification ... 2

Supplement section 2. Detailed costing of primary care consultations ... 2

Supplement section 3. Preparation of covariates ... 3

Supplement section 4. Interactions between co-occurring events during annual periods ... 4

Supplement section 5. Selection of one-part or two-part statistical model ... 4

Supplement section 6. Selection among candidate generalized linear models (GLMs) ... 6

Supplement section 7. Estimation of excess annual costs associated with adverse events ... 8

Supplement section 8. An example of annual primary and hospital care costs calculations using the models ... 8

Supplement tables ... 13

Supplement table 1. Main code lists for adverse events' identification ... 13

Supplement table 2. Unit costs for diagnostic and monitoring tests ... 14

Supplement table 3. Baseline characteristics of participants included in hospital care costs analysis contributing and not contributing to primary care costs analysis. ... 15

Supplement table 4. Adverse events and healthcare use during study follow-up ... 17

Supplement table 5. Excess annual hospital costs associated with adverse events by sex and by age (50 and 60 years old). ... 18

Supplement figures ... 19

Supplement figure 1. A flowchart of inclusion of UK Biobank participants in annual primary and hospital care costs analyses ... 19

Supplementary appendix references ... 20

Supplement sections

Supplement section 1. Adverse events identification

Myocardial infarctions (MIs) and strokes were identified based on the International Classification of Diseases Tenth Revision (ICD10) diagnostic codes (Supplement table 1) using the earliest date identified from the linked primary care, hospital admission and death registry records. Coronary revascularizations (CRVs) were identified based on the Operating Procedure Codes Supplement Fourth Revision (OPCS4) procedural codes using hospital admission records. Incident diabetes cases were identified using the earliest diabetes diagnosis codes in primary care, hospital admission, death records or date of anti-diabetic treatment from the primary care records. Incident cancers were identified using the respective ICD10 codes in the cancer registry records. Deaths were identified using the death registry records, with primary cause of deaths related to circulatory system assigned to vascular death (VD) and other primary causes to non-vascular death (NVD).

Supplement section 2. Detailed costing of primary care consultations

Primary care consultations were costed using an average cost per consultation based on the joint distribution of the type of staff providing the consultation (staff type) and the type of consultation (consultation type), using the unit cost per minute by staff type, and the average duration of consultation by staff type and consultation type.

The joint distribution of staff type and consultation type was derived from Hobbs et al [1]. Hobbs et al reported the annual consultation rate by staff type (GP or nurse) and consultation type (face-to-face, telephone, or home visit) across 100 million consultations in England from 2007 to 2014. The same categories of staff type and consultation type as Hobbs et al were used in this study. The reported rates across 2013 to 2014 were used to generate the proportions for each category.

The unit costs by staff type were derived from the Unit Costs of Health and Social Care 2019/2020[2] that reported unit costs per minute of GP's time; and of GP practice nurse. The unit costs excluding qualification costs were used (GP: £3.10; nurse: £0.63).

The duration of consultation by staff type and consultation type was derived from the GP workload survey 2006/2007[3]. The face to face consultation was assumed to be the same as surgery consultation reported in the survey. The average duration of consultation for all GPs (partner, salaried and registrar) was used for GP, and the average duration of consultation for all other clinical staff (practice nurse, nurse practitioner, and midwife etc.) was used for nurse.

Table SS1 presents the unit costs used, duration and distribution by category of consultations. The average cost per consultation was estimated at £29.01.

Table SS1. Distribution by category of consultations, unit cost and duration of consultations

Consultation	Share of all consultations	Cost (per minute, £2020)	Average duration (minutes)
**GP**			
Face-to-Face	61.63%	3.10	11.9
Telephone	10.47%	3.10	7.4
Home visit	1.55%	3.10	25.9
**Nurse**			
Face-to-Face	24.61%	0.63	15.9
Telephone	1.36%	0.63	9.0
Home visit	0.39%	0.63	36.3

Supplement section 3. Preparation of covariates

Missing data imputation

For a small number of patients, data was missing about their diet quality, ethnicity, smoking status, blood pressure, body mass index (BMI), creatinine, high density lipoprotein (HDL) cholesterol and low density lipoprotein (LDL) cholesterol. Missing ethnicities were assigned to the majority white ethnicity. Missing smoking statuses were assigned to the majority status by sex, age and education. Missing diet qualities were assigned to unhealthy, since the exploratory analysis using diet quality with missing values as a separate category in a Cox regression model indicated similar association for missing as for unhealthy diet quality. Missing Townsend scores were imputed by regressing it on index of multiple deprivation (IMD) scores, years and sources (England, Wales or Scotland) if IMD scores were available, and imputed with ward-level Townsend score with participants' ordnance survey coordinates if IMD scores were missing. Other missing data including missing HbA1c (for identification of baseline diabetes) were imputed using the average imputed values from 20 multiple imputations with chained equations including age, sex, (imputed) ethnicity, (imputed) smoking status, baseline cardiovascular diseases, treated hypertension, statin treatment status and baseline diabetes as auxiliary variables[4]. Due to large proportion of missing values in physical activity (19.4%), missing physical activities were assigned to a separate "Missing value" level.

Covariate specification

Continuous covariates were centred, including age, HDL cholesterol, LDL cholesterol, creatinine, systolic blood pressure, and diastolic blood pressure. Of them HDL cholesterol and creatinine were centred after taking natural logarithm of their value to account for the skewness in their distributions. For unordered discrete covariates, reference levels were assigned as follows: sex (male), ethnicity (white) and prior cardiovascular disease (CVD: myocardial infarction (MI) only, for models in people with previous CVD). For ordinal covariates, reference level was assigned to the 'healthier' category: smoking (never), diet quality (healthy), BMI (≥18.5, <25), treatment/disease ("no" for on antihypertensive treatment, type 1 diabetes, and severe mental illness), year with adverse events ("no" for MI, stroke, coronary revascularisation (CRV), vascular death, incident diabetes, incident cancer and non-vascular death); or the middle level: Townsend socioeconomic deprivation (quintile 3), physical activity (moderate).

For each temporal adverse event history covariate, the consecutive years since event were merged if similar sized associations with annual costs were observed. Firstly, temporal adverse event history covariates were considered as categorical covariates using each year since adverse event as a unique level in the category, and were included in a linear regression for annual costs with the other non-event candidate covariates. Consecutive years/levels since adverse event with similar effects (judged using F-test at 1% significance level) were combined. The cost impacts were hypothesised to decrease with duration from MI, stroke, CRV and cancer, with the temporal histories tested from least to most recent. A previous study [5] showed significant increases in healthcare costs due to diabetes complications over time, such as cardiovascular disease, amputation, blindness, cataract extraction, however these complications take time to develop [6]. Therefore, the temporal diabetes histories covariate was set to three categories variable (no, within 10 years and more than 10 years), and diabetes duration categories were tested for further combination.

Supplement section 4. Interactions between co-occurring events during annual periods

Interactions between co-occurring events during the annual periods were investigated after the completion of model selection (type of model and covariates). Interactions between co-occurring events were considered for inclusion in the model for all pairs of adverse events meeting the following criteria:

1. The number of cases when both events happen in the same year is more than 50
2. The number of cases when both events happen in the same year is more than 5% of the total number of each of separate events in the analysis

For each candidate pair of adverse events, new covariates were created representing interactions in the year of events only.

Supplement section 5. Selection of one-part or two-part statistical model

The proportion of non-zero annual primary care costs was 92.7% across participants without previous CVD and 97.9% across participants with previous CVD. The distribution of the annual primary care costs was similar before and after the exclusion of the years with zero cost, both were right skewed with long right tail in the direction of higher costs. Therefore, annual primary care costs were analysed using one-part generalized linear models (GLMs).

The proportion of non-zero annual hospital costs was 15.8% across participants without previous CVD, and 28.4% across participants with previous CVD. The distribution of the annual hospital care costs was significantly different before and after the exclusion of years with zero cost. After the exclusion of years with zero cost, the distributions were right skewed with long right tail in the direction of higher costs. Therefore, the annual hospital costs were analysed using the two-part model, with the first part modelling the probability of having any positive costs using logistic regression, and the second part modelling the costs conditional on any positive costs using GLMs. (Figure SS4)

Figure SS4. Distribution of annual primary care costs (A: excluding costs > £3000, 1% of total) and annual hospital care costs (B: excluding costs > £10000, 1% of total)

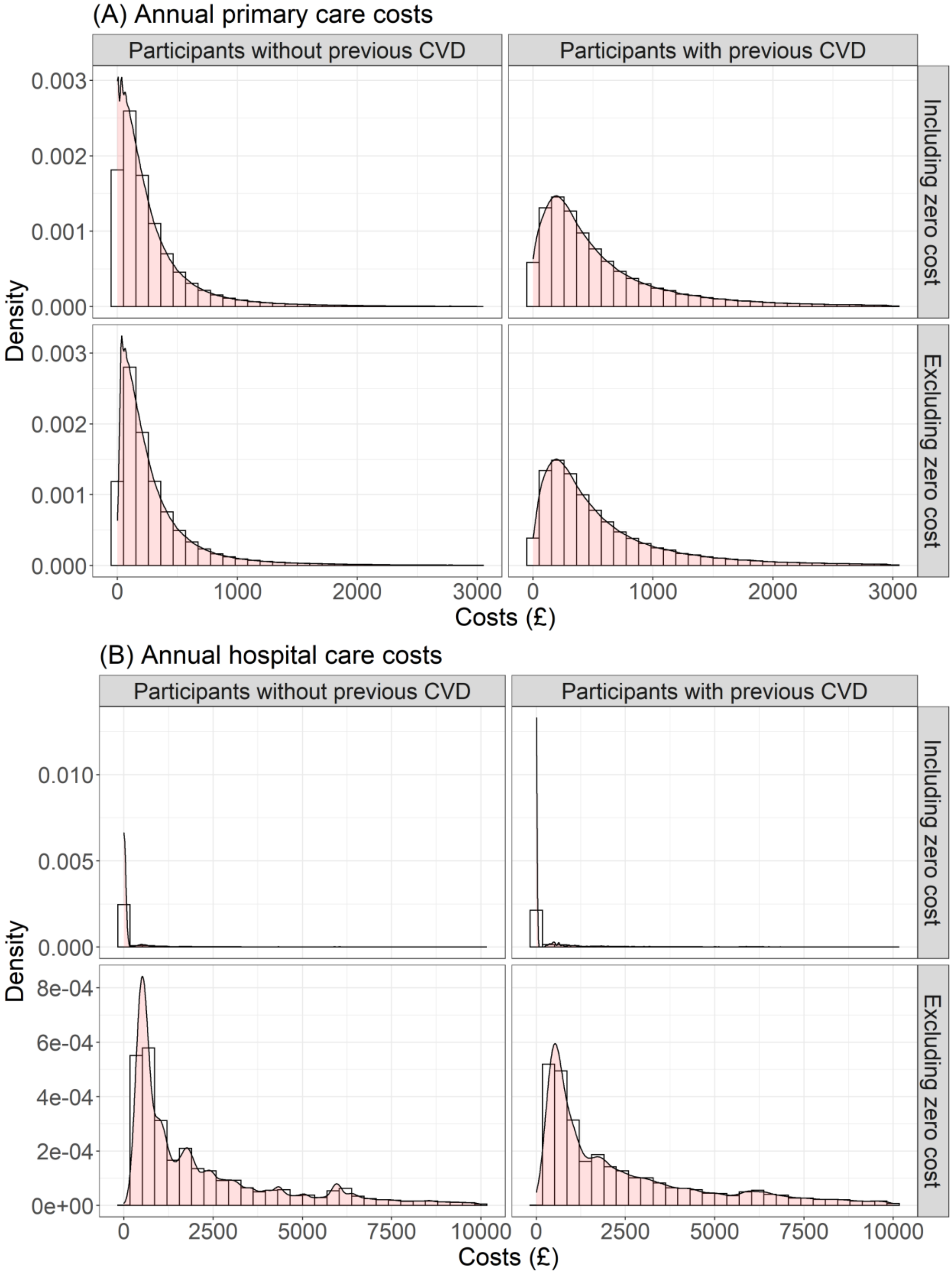

Supplement section 6. Selection among candidate generalized linear models (GLMs)

Six different GLMs using three distributions (Gaussian, Poisson, and Gamma) and two link functions (Identity and natural log) were considered for the primary care costs models and for the second part of the hospital costs models. Stepwise bidirectional covariate selection were performed for all GLM models at 1% level of significance. The GLMs with the selected covariates were compared in terms of specification and performance tests. The appropriateness of family distribution and link function was assessed using three specification tests: Modified Park's test[7], Hosmer-Lemeshow test[8], and Pregibon's link test[9]. The model performance was assessed with three measures: mean error, mean absolute error and root mean squared error.

In both primary and hospital care costs models in both those with and without previous CVD, the slope coefficients from the Modified Park's tests ranged from 1 to 2 suggesting the use of either Poisson (slope = 1) or Gamma distribution (slope = 2) for the variance functions in the costs models. As the developed models were based on large sample size, Hosmer-Lemeshow test and Pregibon's test in most models were significant, not providing direct information on which link function is better. However, in the hospital costs models in people with previous CVD, Hosmer-Lemeshow tests in the GLMs using identity link were all not significant indicating identity link to be a better link function compared to log link; in all the models, the absolute value of the estimated coefficients for the Pregibon's test in the GLMs using identity link were all less than 0.001, but those in the GLMs using log link were all higher than 0.11, also indicating identity link a better link function. There were only minor differences in performance between models across the different GLMs (Table SS5). By plotting the mean error by decile of predicted annual costs, the GLM with Poisson distribution and identity link performed better across different decile of predicted annual costs compared to the other GLMs for both primary and hospital care costs in both populations (Figure SS5). Therefore, the GLM with Poisson distribution and identity link was used to model the primary care costs and also the second part of the hospital care costs.

Table SS5. Tests of GLM model specification and performance

GLM model	Model specification test			Model performance test		
	Modified Park's test	Hosmer-Lemeshow test	Pregibon's test	ME	MAE	RMSE
Annual primary care cost (One-part GLM model)						
Participants without previous CVD						
Gaussian – Identity	NA^	<0.001*	<0.001*[&]	0	259	737
Gaussian – LOG	1.563	<0.001*	<0.001*	-7	263	738
Poisson – Identity	1.218	<0.001*	<0.001*[&]	-6	262	739
Poisson – LOG	1.364	<0.001*	<0.001*	0	259	740
Gamma – identity	1.236	<0.001*	<0.001*[&]	8	257	739
Gamma – LOG	1.211	<0.001*	<0.001*	-9	264	747
Participants with previous CVD						
Gaussian – Identity	NA^	<0.001*	0.003[&]	0	499	1309
Gaussian – LOG	1.824	<0.001*	0.020	-7	501	1310
Poisson – Identity	1.479	<0.001*	<0.001*[&]	0	499	1312
Poisson – LOG	1.753	<0.001*	<0.001*	0	496	1311
Gamma – identity	1.708	<0.001*	<0.001*[&]	16	493	1313
Gamma – LOG	1.602	<0.001*	<0.001*	-11	501	1317

GLM model	Model specification test			Model performance test		
	Modified Park's test	Hosmer-Lemeshow test	Pregibon's test	ME	MAE	RMSE
Annual hospital care cost (Part-2 GLM conditional incurring any hospital cost at the year)						
Participants without previous CVD						
Gaussian – Identity	1.754	<0.001*	<0.001*&	0	2505	4578
Gaussian – LOG	1.925	<0.001*	<0.001*	-38	2540	4604
Poisson – Identity	1.774	<0.001*	<0.001*&	0	2504	4579
Poisson – LOG	1.889	<0.001*	<0.001*	0	2518	4615
Gamma – identity	1.770	<0.001*	0.001&	-5	2506	4580
Gamma – LOG	1.802	<0.001*	<0.001*	-32	2530	4659
Participants with previous CVD						
Gaussian – Identity	1.550	0.008	<0.001*&	0	3129	5754
Gaussian – LOG	1.766	<0.001*	<0.001*	-56	3185	5786
Poisson – Identity	1.573	0.040	0.002&	0	3127	5754
Poisson – LOG	1.686	<0.001*	<0.001*	0	3151	5799
Gamma – identity	1.565	0.070	0.071&	-8	3129	5756
Gamma – LOG	1.585	<0.001*	<0.001*	-49	3169	5857

CVD, cardiovascular disease; GLM, generalized linear model; ME, mean error; MAE, mean absolute error; RMSE, root mean squared error; NA, not applicable;

*: Significant difference at 0.1% level;

&: absolute value of the estimated coefficient less than 0.001;

^: Test could not be performed due to negative results were predicted from Gaussian-identity model

Figure SS5. Mean prediction error by decile of predicted costs

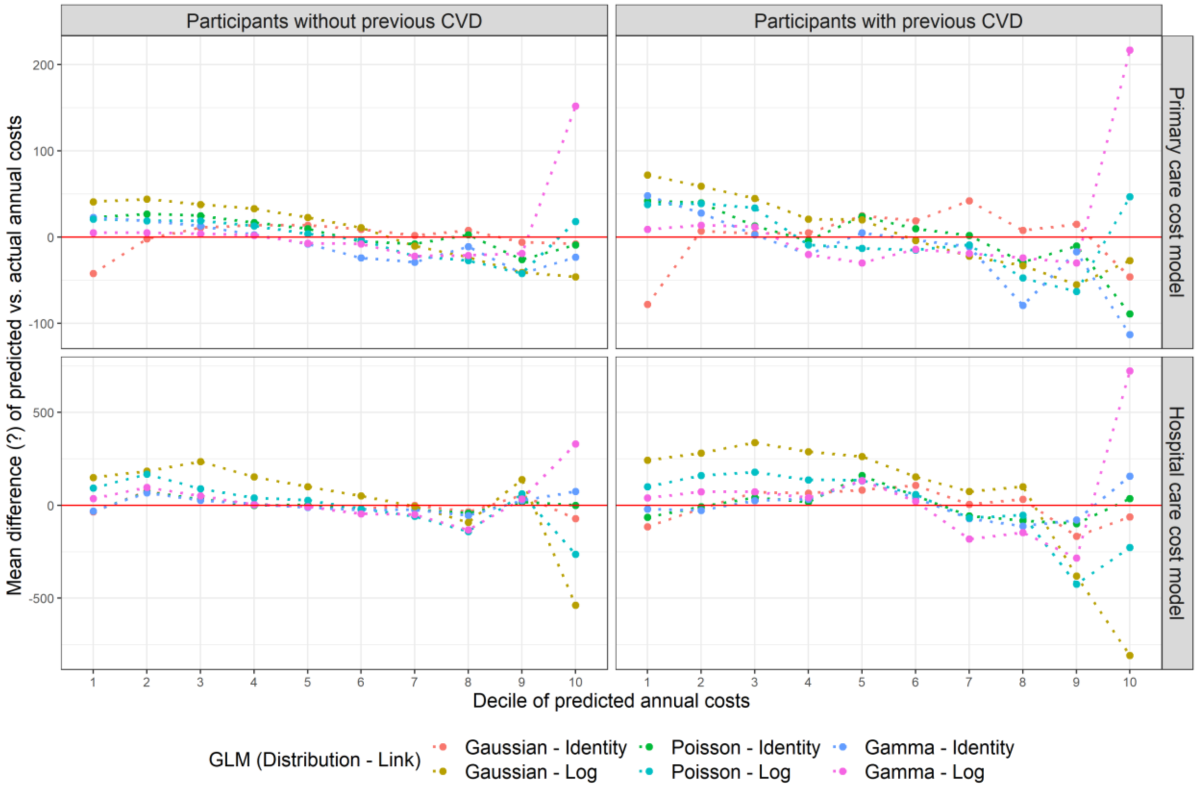

Supplement section 7. Estimation of excess annual costs associated with adverse events

The costs' models were used to derive the marginal effects of adverse events on annual costs. For primary care costs, marginal effects are the same as respective parameter estimates. For hospital costs, the marginal effects were derived using recycled prediction since hospital costs' models were non-linear. The recycled predictions were derived in the following way:

(1) run two scenarios across samples by setting the event history covariate as per the following 2 scenarios
    a. at event history of interest
    b. at no event history
(2) calculate the difference in mean annual costs between these 2 scenarios.

To estimate the marginal effects for an event interacted with other events, all the other pair of the interacted events were set to "no event" as well for the "no event history" scenario.

Standard errors of the mean difference were generated from 1,000 bootstrap replicates.

Supplement section 8. An example of annual primary and hospital care costs calculations using the models

To illustrate the use of the annual primary and hospital care models, we present the numeric calculation of costs for an individual with previous CVD (Stroke only) having the following hypothetical profile: a 50-years old female, of white ethnicity, quintile 1 Townsend socioeconomic deprivation, never smoked, high level of physical activity, healthy diet, normal BMI level (18.5-25 kg/m2), with LDL cholesterol of 4 mmol/L, HDL cholesterol of 2 mmol/L, creatinine of 90 umol/L, SBP of 160 mmHg, DBP of 80 mmHg, not on antihypertensive treatment, without histories of severe mental illness and type 1 diabetes, had a MI in the year, had a CRV 1 year ago, was diagnosed with cancer ≥10 years ago, without other incident cardiovascular or other events modelled. Tables SS8A and SS8B show the calculation for predicted annual primary and hospital care costs using the models in Table 2 and 3, respectively.

Table SS8A. Calculation of annual primary care costs using the primary care costs model

**Covariate**	**Coefficient**	**Covariate value from individual profile**	**Coefficient x Covariate value**
Intercept[a]	302	1	302
**BASELINE CHARACTERISTICS**			
Male (ref: female)	-50	0	0
Ethnicity (ref: white)			
Black	61	0	0
South Asian	24	0	0
Others*	-107	0	0
Townsend socioeconomic deprivation (ref: Quintile 3)			
Quintile 1 (least deprived)	6	1	6
Quintile 2	25	0	0
Quintile 4	49	0	0
Quintile 5	132	0	0

**Covariate**	**Coefficient**	**Covariate value from individual profile**	**Coefficient x Covariate value**
Smoking (ref: never)			
Former smoker	[b]	0	NA
Current smoker	[b]	0	NA
Physical activity (ref: moderate)			
Low	117	0	0
High	-8	1	-8
Missing	96	0	0
BMI (ref: ≥18.5, <25)			
<18.5	182	0	0
≥25, <30	8	0	0
≥30, <35	58	0	0
≥35, <40	194	0	0
≥40	329	0	0
LDL cholesterol (centred at 3.6; per 1 mmol/L)	[b]	4-3.6	NA
Natural logarithm of HDL cholesterol (lnmmol/L)	[b]	Ln(2)	NA
Systolic blood pressure (centred at 140; per 20 mmHg)	-20	(160–140)/20	-20
On antihypertensive treatment (ref: no)	93	0	0
Severe mental illness history (ref: no)	280	0	0
Prior type 1 diabetes (ref: no)	731	0	0
Prior CVD (ref: MI only)			
PAD only	66	0	0
Stroke only	124	1	124
Other CHD only^	84	0	0
Two or more	221	0	0
**TIME-UPDATED CHARACTERISTICS**			
Current Age (centred at 60; per 10 years)	62	(50-60)/10	-62
Incident MI (ref: no)	231	1	231
Incident Stroke (ref: no)	428	0	0
Incident CRV (ref: no)			
Same year	233	0	0
≥1 year ago	10	1	10
Diabetes (ref: no)			
<10 years ago	343	0	0
≥10 years ago	568	0	0
Cancer (ref: no)			
<5 years	236	0	0
≥5, <10 years	123	0	0
≥10 years ago	[c]	1	123
VD (ref = no)	-16	0	0
NVD (ref = no)	198	0	0

Covariate	Coefficient	Covariate value from individual profile	Coefficient x Covariate value
**INTERACTIONS**			
Any incident MI and same year CRV (ref = no)	[b]	0	NA
Any incident MI and same year VD (ref = no)	-500	0	0
Any incident stroke and same year VD (ref = no)	-439	0	0
<5 years cancer and same year NVD (ref = no)	[b]	0	NA
**Predicted annual costs: sum of coefficients x value =**			**706**

[a] Annual cost for an individual in the reference categories of covariates
[b] Covariate or interaction term excluded during the selection procedure (not statistically significant)
[c] Same as the preceding annual category

Table SS8B. Calculation of annual hospital care costs using the hospital costs model

Covariate	Part 1 Exp(Coefficient)	Part 2 Coefficient	Covariate value from individual profile	Coefficient x Covariate value	
				Part 1: Ln[Exp(Coefficient)] x covariate value	Part 2
Intercept [a]	0.19	2326	1	-1.647	2326
**BASELINE CHARACTERISTICS**					
Male (ref: female)	0.87	-125	0	0	0
Ethnicity (ref: white)					
Black	1.06	-412	0	0	0
South Asian	1.21	-426	0	0	0
Others*	1.09	-246	0	0	0
Townsend socioeconomic deprivation (ref: Quintile 3)					
Quintile 1 (least deprived)	0.91	[b]	1	-0.091	NA
Quintile 2	0.95	[b]	0	0	NA
Quintile 4	1.06	[b]	0	0	NA
Quintile 5	1.15	[b]	0	0	NA
Smoking (ref: never)					
Former smoker	1.06	8	0	0	0
Current smoker	1.13	276	0	0	0
Physical activity (ref: moderate)					
Low	1.25	415	0	0	0
High	1.04	-13	1	0.043	-13
Missing	1.17	156	0	0	0
Unhealthy diet (ref: Healthy diet)	1.06	[b]	0	0	NA
Body mass index (kg/m^2) (ref: ≥18.5, <25)					
<18.5	1.43	1007	0	0	0
≥25, <30	1.04	16	0	0	0
≥30, <35	1.14	177	0	0	0
≥35, <40	1.21	381	0	0	0

≥40	1.34	840	0	0	0
LDL cholesterol (centred at 3.6; per 1 mmol/L)	[b]	[b]	4-3.6	NA	NA
Natural logarithm of HDL cholesterol (lnmmol/L)	[b]	[b]	Ln(2)	NA	NA
Natural logarithm of creatinine centred at 4.4; per 0.2 lnumol/L	1.02	107	(Ln(90) – 4.4)/0.2	0.012	53
Systolic blood pressure (centred at 140; per 20 mmHg)	0.95	[b]	(160-140) /20	-0.050	NA
Diastolic blood pressure (centred at 80; per 10 mmHg)	[b]	[b]	0	NA	NA
On antihypertensive treatment (ref: no)	1.11	[b]	0	0	NA
Severe mental illness history (ref: no)	1.39	227	0	0	0
Prior type 1 diabetes (ref: no)	1.69	792	0	0	0
Prior CVD (ref: MI only)					
PAD only	1.19	498	0	0	0
Stroke only	1.11	113	1	0.106	113
Other CHD only^	1.27	105	0	0	0
Two or more	1.43	381	0	0	0
**TIME-UPDATED CHARACTERISTICS**					
Current age (centred at 60; per 10 years)	1.25	121	(50-60)/10	-0.220	-121
Incident MI (ref: no)					
Same year	47.33	3965	1	3.857	3965
1 year ago	1.71	1011	0	0	0
2 years ago	1.28	696	0	0	0
≥3 years ago	[c]	[c]	0	0	0
Incident Stroke (ref: no)					
Same year	46.65	4591	0	0	0
1 year ago	2.19	1561	0	0	0
2 years ago	1.52	[c]	0	0	0
≥3 years ago	[c]	[c]	0	0	0
Incident CRV (ref: no)					
Same year	[d]	5117	0	0	0
1 year ago	1.54	599	1	0.431	599
2 years ago	1.32	4	0	0	0
≥3 years ago	[c]	[c]	0	0	0
Diabetes (ref: no)					
<10 years ago	1.36	408	0	0	0
≥10 years ago	1.22	[c]	0	0	0
Cancer (ref: no)					
Same year	24.59	5160	0	0	0
1 year ago	3.81	3475	0	0	0
2 years ago	2.39	1863	0	0	0
3 years ago	2.09	1601	0	0	0
4 years ago	1.92	947	0	0	0
≥5 years ago	1.6	[c]	1	0.467	947
VD (ref = no)	2.38	4749	0	0	0

NVD (ref = no)	9.1	6412	0	0	0
**EVENT INTERACTIONS**					
Same year MI and same year CRV (ref = no)	[d]	-3358	0	0	0
Same year MI and same year VD (ref = no)	0.02	-4874	0	0	0
Same year Stroke and same year VD (ref = no)	0.09	-4308	0	0	0
Same year cancer and same year NVD (ref = no)	0.37	-1529	0	0	0
**Predicted results at each part: sum of coefficients x value**<br>**$Odds_{p1}$=Exp(sum of $coefficients_{p1}$ x $value_{p1}$)**				**2.908**<br>**Exp(2.908)= 18.3**	**7869**
**Predicted annual costs: ($Odds_{P1}$ / (1+$Odds_{P1}$) * $Costs_{P2}$) = 18.3/(1+18.3) * 7869 = 7462**					

[a] Values for an individual in the reference categories of covariates (odds for part 1 model and cost for part 2 model)
[b] Covariate was excluded during the selection procedure (not statistically significant)
[c] Same as the preceding annual category
[d] Incurring cost is certain in annual periods with CRV.

Supplement tables

Supplement table 1. Main code lists for adverse events' identification

Adverse events	Type of code	Included code
**Cardiovascular events**		
Myocardial infarction	ICD10	I21, I22, I23, or I241
Stroke	ICD10	I60, I61, I62, I63, or I64
Coronary revascularization	OPCS4	K40, K41, K42, K43, K44, K45, K46, K49, K501, K504, K75, or K76
Vascular death	ICD10	Primary reason of death to be from chapters I, R; or Primary reason of death to be Y832, Y835 or W19 with secondary reason of death to be from chapter I
**Non-vascular events**		
Diabetes	ICD10	E10, E11, E13, or E14
Cancer	ICD10	All chapter C except C44
Non-vascular death	ICD10	Death not considered as vascular death

Supplement table 2. Unit costs for diagnostic and monitoring tests

Category for costing	Included read code/s	Cost, £	Source code
Diagnostic procedures			
Neuropsychology testing	311	145.23	DADS weighted across all AA
Auditory testing	313	86.30	DADS weighted across all CA37
ECG	32	49	DADS EY51Z
Echocardiogram	33B	85	DADS EY50Z
Biopsy	3B	406.94	DADS weighted across YC01Z, YG12Z, YJ09Z, YJ13Z
Endoscopy	36	381.66	OPROC weighted across all FE0 to FE4
Laboratory test			
Hematology	42	3	DAPS05
Immunology	43	7	DAPS06
Blood chemistry	44	1	DAPS04
Microbiology	4J	8	DAPS07
Cytology or Histology	4K	12.54	Weighted by DAPS01 and DAPS02
Other pathology	46, 47, 48, 49, 4A, 4B, 4C, 4D, 4E, 4F, 4G, 4H, 4L, 4M, 4N, 4P	6	DAPS09
Diagnostic imaging			
X ray	52, 53, 56	31	DADS DAPF
CAT	54	144.79	IMAGDA weighted across all RD3
Arteriography	55	103.19	DADS YR25Z
MRI	57	142.37	IMAGDA weighted across all RD0
Ultrasound Scan	58	55.94	IMAGDA weighted across all RD4

CAT, computerized tomography; ECG, electrocardiogram; MRI, magnetic resonance imaging.
DADS, Directly Accessed Diagnostic Services; DAPF, Direct Access Plain Film; DAPS, Directly Accessed Pathology Services; IMAGDA, Imaging: Direct Access; OPROC, Outpatient Procedures.

Supplement table 3. Baseline characteristics of participants included in hospital care costs analysis contributing and not contributing to primary care costs analysis.

	**Included in primary care costs analysis**		**Excluded from primary care costs analysis**	
	**Without previous CVD (n = 168 205)**	**With previous CVD (n = 24 778)**	**Without previous CVD (n = 276 331)**	**With previous CVD (n = 32 493)**
Age (years)	56.1 (8.0)	60.3 (7.1)	56.0 (8.1)	60.5 (7.0)
Male	73 573 (43.7)	14 084 (56.8)	121 406 (43.9)	19 645 (60.5)
Ethnicity				
White	159 517 (94.8)	23 570 (95.1)	258 447 (93.5)	30 551 (94)
Black	1 851 (1.1)	222 (0.9)	5 415 (2)	548 (1.7)
South Asian	2 905 (1.7)	495 (2)	4 078 (1.5)	563 (1.7)
Other*	3 170 (1.9)	355 (1.4)	6 742 (2.4)	607 (1.9)
Missing	762 (0.5)	136 (0.5)	1 649 (0.6)	224 (0.7)
Townsend socioeconomic deprivation				
Quintile 1 (least deprived)	52 210 (31)	6 571 (26.5)	86 579 (31.3)	8 438 (26)
Quintile 2	37 736 (22.4)	5 223 (21.1)	60 414 (21.9)	6 499 (20)
Quintile 3	29 506 (17.5)	4 397 (17.7)	46 164 (16.7)	5 476 (16.9)
Quintile 4	25 853 (15.4)	4 132 (16.7)	42 519 (15.4)	5 377 (16.5)
Quintile 5	18 554 (11)	3 809 (15.4)	33 752 (12.2)	5 882 (18.1)
Missing	4 346 (2.6)	646 (2.6)	6 903 (2.5)	821 (2.5)
Smoking				
Never	94 763 (56.3)	11 069 (44.7)	153 533 (55.6)	13 811 (42.5)
Former smoker	55 607 (33.1)	10 654 (43)	92 174 (33.4)	14 344 (44.1)
Current smoker	17 016 (10.1)	2 874 (11.6)	28 963 (10.5)	4 053 (12.5)
Missing	819 (0.5)	181 (0.7)	1 661 (0.6)	285 (0.9)
Physical activity				
Low	24 777 (14.7)	4 265 (17.2)	41 144 (14.9)	5 839 (18)
Moderate	55 199 (32.8)	7 676 (31)	90 947 (32.9)	10 001 (30.8)
High	55 680 (33.1)	7 468 (30.1)	89 512 (32.4)	9 308 (28.6)
Missing	32 549 (19.4)	5 369 (21.7)	54 728 (19.8)	7 345 (22.6)
Diet quality				
Healthy	108 313 (64.4)	15 553 (62.8)	177 676 (64.3)	20 017 (61.6)
Unhealthy	56 945 (33.9)	8 643 (34.9)	92 132 (33.3)	11 523 (35.5)
Missing	2 947 (1.8)	582 (2.3)	6 523 (2.4)	953 (2.9)
Body mass index (kg/m^2)				
<18.5	840 (0.5)	128 (0.5)	1 524 (0.6)	125 (0.4)
≥18.5, <25	55 226 (32.8)	6 035 (24.4)	93 620 (33.9)	7 317 (22.5)
≥25, <30	71 515 (42.5)	10 344 (41.7)	116 442 (42.1)	13 530 (41.6)
≥30, <35	28 779 (17.1)	5 485 (22.1)	45 617 (16.5)	7 552 (23.2)
≥35, <40	7 949 (4.7)	1 802 (7.3)	12 696 (4.6)	2 510 (7.7)
≥40	2 990 (1.8)	733 (3)	4 881 (1.8)	1 080 (3.3)
Missing	906 (0.5)	251 (1)	1 551 (0.6)	379 (1.2)

	Included in primary care costs analysis		Excluded from primary care costs analysis	
	Without previous CVD (n = 168 205)	With previous CVD (n = 24 778)	Without previous CVD (n = 276 331)	With previous CVD (n = 32 493)
LDL cholesterol (mmol/L)	3.6 (0.9)	3.1 (0.9)	3.6 (0.8)	3.1 (0.9)
HDL cholesterol (mmol/L)	1.5 (0.4)	1.3 (0.4)	1.5 (0.4)	1.3 (0.4)
Creatinine (umol/L)	71.5 (15.1)	76.5 (19.5)	71.5 (15.0)	77.4 (20.1)
Systolic blood pressure (mmHg)	138.2 (18.7)	139.1 (19.0)	137.5 (18.6)	138.7 (18.9)
Diastolic blood pressure (mmHg)	82.6 (10.1)	81.0 (10.4)	82.3 (10.1)	80.8 (10.5)
On antihypertensive treatment	27 240 (16.2)	10 900 (44)	44 685 (16.2)	15 281 (47)
Prior diabetes				
Type 1	926 (0.6)	558 (2.3)	1 561 (0.6)	820 (2.5)
Type 2	7 134 (4.2)	2 694 (10.9)	11 941 (4.3)	4 098 (12.6)
Prior cancer	12 221 (7.3)	2 420 (9.8)	20 491 (7.4)	3 439 (10.6)
Severe mental illness history	17 549 (10.4)	3 374 (13.6)	18 533 (6.7)	2 949 (9.1)
Prior CVD				
No	168 205 (100)	0 (0)	276 331 (100)	0 (0)
MI only	0 (0)	776 (3.1)	0 (0)	1 294 (4)
Stroke only	0 (0)	1 991 (8)	0 (0)	3 146 (9.7)
PAD only	0 (0)	3 473 (14)	0 (0)	3 332 (10.3)
Other CHD only^	0 (0)	12 642 (51)	0 (0)	16 327 (50.2)
Two or more	0 (0)	5 896 (23.8)	0 (0)	8 394 (25.8)

CHD, coronary heart disease; CVD, cardiovascular disease; HDL, high density lipoprotein; LDL, low density lipoprotein; PAD, peripheral arterial disease; SD, standard deviation.
Values are mean (SD) or number (%).
*Other ethnicity includes Chinese, Mixed, White and Black Caribbean, White and Black African, White and Asian, Any other mixed background and other ethnic group.
^Other CHD includes acute rheumatic fever, chronic rheumatic heart diseases, hypertensive heart disease, angina pectoris, other acute ischaemic heart disease, chronic ischaemic heart disease, pulmonary heart disease and other form of heart disease.

Supplement table 4. Adverse events and healthcare use during study follow-up

	Primary care costs analysis		Hospital care costs analysis	
	**Without previous CVD (n = 168 205)**	**With previous CVD (n = 24 778)**	**Without previous CVD (n = 444 536)**	**With previous CVD (n = 57 271)**
Duration of follow-up (years)	7.1 (0.9)	7.0 (1.2)	7.1 (1.0)	7.0 (1.3)
Number of participants with events during follow-up				
Myocardial infarction	1 841 (1.1)	871 (3.5)	4 651 (1.0)	2 210 (3.9)
Stroke	1 730 (1.0)	812 (3.3)	4 106 (0.9)	1 872 (3.3)
Coronary revascularization	2 197 (1.3)	1 205 (4.9)	5 877 (1.3)	3 094 (5.4)
Incident diabetes[&]	3 425 (2.1)	1 077 (5.0)	7 395 (1.7)	2 301 (4.7)
Incident cancer[&]	9 057 (5.8)	1 797 (8.0)	25 376 (6.2)	4 498 (8.7)
Vascular death	521 (0.3)	457 (1.8)	1 781 (0.4)	1 412 (2.5)
Non-vascular death	2 777 (1.7)	869 (3.5)	9 067 (2.0)	2 632 (4.6)
Healthcare use during follow-up				
Total number of person-years used for analysis	1 096 034	159 707	3 371 754	426 570
Person- years with primary /hospital care costs, respectively	1 015 858 (92.7)	156 379 (97.9)	534 287 (15.8)	121 167 (28.4)
Number of primary care consultations/ hospital inpatient episodes, respectively, per person-year (95% CIs)	5.10 (5.08, 5.11)	8.31 (8.24, 8.38)	0.34 (0.33, 0.34)	0.71 (0.70, 0.72)
Number of diagnostic and monitoring tests per person-year (95% CIs)	2.98 (2.96, 2.99)	5.84 (5.77, 5.91)		
Number of prescription medications per person-year (95% CIs)	17.9 (17.8, 18.0)	48.3 (47.6, 49.0)		
Annual cost (£) per person-year				
Total primary/ hospital inpatient care costs, respectively	360 (356, 363)	746 (730, 762)	514 (510, 518)	1 131 (1 114, 1 149)
Primary care consultations	148 (147, 148)	241 (239, 243)		
Diagnostic and monitoring tests (primary care)	28 (28, 28)	49 (48, 49)		
Prescription medications (primary care)	183 (180, 187)	456 (441, 471)		

CVD, cardiovascular disease; CI, confidence interval; SD, standard deviation.
Values are mean (SD) or number (%) unless stated otherwise.
[&]Calculated as proportion from participants without condition at baseline.

Supplement table 5. Excess annual hospital costs associated with adverse events by sex and by age (50 and 60 years old).

Event (Reference: No event)	Participants without previous CVD		Participants with previous CVD		Participants without previous CVD		Participants with previous CVD	
	**Male**	**Female**	**Male**	**Female**	**Age 50**	**Age 60**	**Age 50**	**Age 60**
Cardiovascular events								
Myocardial infarction								
Same year	4310	4370	5580	5640	4150	4380	5520	5600
1 year ago	350	380	690	750	290	370	600	680
2 years ago	180	190	360	390	150	190	310	350
≥3 years ago	150	170	[a]	[a]	130	160	[a]	[a]
Stroke								
Same year	5550	5630	6140	6200	5350	5630	6080	6160
1 year ago	1020	1080	1140	1230	860	1070	1010	1140
2 years ago	400	420	780	850	330	410	680	780
≥3 years ago	300	320	[a]	[a]	250	320	[a]	[a]
Coronary revascularization								
Same year	7120	7150	7200	7200	7100	7160	7240	7220
1 year ago	210	220	460	500	170	220	400	460
2 years ago	170	180	160	170	140	170	140	160
≥3 years ago	120	120	[a]	[a]	90	120	[a]	[a]
Vascular death	1510	1600	2640	2830	1260	1580	2330	2620
Non-vascular events								
Diabetes								
<10 years ago	150	160	300	330	120	160	260	300
≥10 years ago	80	90	230	250	70	90	200	230
Cancer								
Same year	6090	6200	6190	6310	5830	6190	6020	6200
1 year ago	2900	3030	2660	2830	2520	3010	2400	2650
2 years ago	1130	1200	1300	1400	930	1180	1140	1290
3 years ago	820	870	1050	1140	670	860	920	1050
4 years ago	650	690	730	790	530	680	630	720
≥5 years ago	400	430	580	630	320	420	500	570
Non-vascular death	5250	5420	5940	6170	4740	5410	5580	5930

CVD, cardiovascular disease;

Estimates adjusted for patient sociodemographic and clinical characteristics and rounded to 10 UK£.

[a] Excess annual cost remains as in preceding annual period (e.g. £360 excess annual hospital costs in each year after the 2nd year following MI in male with previous CVD).

Supplement figures

Supplement figure 1. A flowchart of inclusion of UK Biobank participants in annual primary and hospital care costs analyses

UK Biobank participant
Participants = 502 412

↓

Excluding participants with end stage renal disease
Participants = 501 807

For primary care cost analysis

With linked primary care records and annual periods without registration gaps
Participants = 192 983

↓

People without previous CVD
Number of participants = 168 205
Person-years of follow-up = 1 191 348
Number of annual periods of follow-up = 1 274 099
Number of annual periods of follow-up without gaps* (% of total) = 1 096 034 (86.0%)

People with previous CVD
Number of participants = 24 778
Person-years of follow-up = 172 761
Number of annual periods of follow-up = 184 983
Number of annual periods of follow-up without gaps* (% of total) = 159 707 (86.3%)

For hospital care cost analysis

With linked hospital admission records
Participants = 501 807

↓

People without previous CVD
Number of participants = 444 536
Person-years of follow-up = 3 157 572
Number of annual periods of follow-up = 3 371 754
Number of annual periods of follow-up without gaps* (% of total) = 3 371 754 (100%)

People with previous CVD
Number of participants = 57 271
Person-years of follow-up = 398 640
Number of annual periods of follow-up = 426 570
Number of annual periods of follow-up without gaps* (% of total) = 426 570 (100%)

*Some participants may move to a primary care practice not contributing linked data to UK Biobank and would, therefore, have gaps in their registration records in the linked primary care data. Their annual periods with gaps in the primary care registrations were excluded from the primary care cost analysis.

Supplementary appendix references

1. Hobbs FDR, Bankhead C, Mukhtar T, et al. Clinical workload in UK primary care: a retrospective analysis of 100 million consultations in England, 2007-14. *Lancet* 2016; **387**(10035): 2323-30.
2. Personal Social Services Research Unit (PSSRU). Unit Costs of Health and Social Care 2020 - Community-based Health Care Staff. 2020. https://www.pssru.ac.uk/pub/uc/uc2020/2-communityhcstaff.pdf (accessed 2021/09/30).
3. NHS Digital. GP workload survey 2006/07. 2007. https://digital.nhs.uk/data-and-information/areas-of-interest/workforce/gp-workload-survey (accessed 2021/09/30).
4. van Buuren S, Groothuis-Oudshoorn K. mice: Multivariate Imputation by Chained Equations in R. *Journal of Statistical Software* 2011; **45**(3): 1-67.
5. Alva ML, Gray A, Mihaylova B, Leal J, Holman RR. The impact of diabetes-related complications on healthcare costs: new results from the UKPDS (UKPDS 84). *Diabet Med* 2015; **32**(4): 459-66.
6. Leal J, Alva M, Gregory V, et al. Estimating risk factor progression equations for the UKPDS Outcomes Model 2 (UKPDS 90). *Diabet Med* 2021; **38**(10): e14656.
7. Manning WG, Mullahy J. Estimating log models: to transform or not to transform? *J Health Econ* 2001; **20**(4): 461-94.
8. Hosmer DW, Lemeshow S. Goodness of fit tests for the multiple logistic regression model. *Commun Stat a-Theor* 1980; **9**(10): 1043-69.
9. Pregibon D. Goodness of link tests for generalized linear models. *Applied Statistics* 1980; **29**: 15-24.
